# Early career retention of Malawian medical graduates: a retrospective cohort study

**DOI:** 10.1111/tmi.12408

**Published:** 2014-10-20

**Authors:** Kate L. Mandeville, Godwin Ulaya, Mylene Lagarde, Lyson Gwesele, Titha Dzowela, Kara Hanson, Adamson S. Muula

**Affiliations:** ^1^ Department of Global Health and DevelopmentLondon School of Hygiene and Tropical MedicineLondonUK; ^2^ Mwanza District HospitalMwanzaMalawi; ^3^ Queen Elizabeth Central HospitalBlantyreMalawi; ^4^ EU Migration Project on Strengthening Specialised Medical Care in MalawiLilongweMalawi; ^5^ Department of Community HealthUniversity of MalawiBlantyreMalawi

**Keywords:** human resources for health, retention, medical education, Malawi, doctors, health policy, ressources humaines pour la santé, rétention, éducation médicale, Malawi, médecins, politique de santé

## Abstract

**Objective:**

There have been longstanding concerns over Malawian doctors migrating to high‐income countries. Early career is a particularly vulnerable period. After significant policy changes, we examined the retention of recent medical graduates within Malawi and the public sector.

**Methods:**

We obtained data on graduates between 2006 and 2012 from the University of Malawi College of Medicine and Malawi Ministry of Health. We utilised the alumni network to triangulate official data and contacted graduates directly for missing or uncertain data. Odds ratios and chi‐squared tests were employed to investigate relationships by graduation year and gender.

**Results:**

We traced 256 graduates, with complete information for more than 90%. Nearly 80% of registered doctors were in Malawi (141/178, 79.2%), although the odds of emigration doubled with each year after graduation (odds ratio = 1.98, 95% CI = 1.54–2.56, *P* < 0.0001). Of the 37 graduates outside Malawi (14.5%), 23 (62.2%) were training in South Africa under a College of Medicine sandwich programme. More than 80% of graduates were working in the public sector (185/218, 82.6%), with the odds declining by 27% for each year after graduation (odds ratio = 0.73, 95% CI = 0.61–0.86, *P* < 0.0001).

**Conclusions:**

While most doctors remain in Malawi and the public sector during their early careers, the odds of leaving both increase with time. The majority of graduates outside Malawi are training in South Africa under visa restrictions, reflecting the positive impact of postgraduate training in Malawi. Concerns over attrition from the public sector are valid and require further exploratory work.

## Abstract (fr)

**Objectif:**

Il existe des préoccupations de longue date à propos de la migration des médecins malawites vers les pays à revenus élevés. Le début de carrière est une période particulièrement délicate. Après des changements de politique importants, nous avons examiné la rétention des récents diplômés en médecine au Malawi et dans le secteur public.

**Méthodes:**

Nous avons obtenu les données sur les diplômés entre 2006 et 2012, du Collège de Médecine de l'Université du Malawi et du Ministère de la Santé du Malawi. Nous avons utilisé le réseau des anciens étudiants pour trianguler les données officielles et avons contacté directement les diplômés pour les données manquantes ou incertaines. Les rapports des cotes (odds ratios) et tests de chi carré ont été utilisés pour étudier les associations par année de diplôme et par sexe.

**Résultats:**

Nous avons retrouvé les traces de 256 diplômés avec des informations complètes pour plus de 90% d'entre eux. Près de 80% des médecins étaient inscrits au Malawi (141/178, 79,2%) bien que les chances pour l’émigration doublaient pour chaque année après l'obtention du diplôme (odds ratio = 1,98; IC95%: 1,54 à 2,56; p <0,0001). Sur les 37 diplômés à l'extérieur du Malawi (14,5%), 23 (62,2%) étaient en formation en Afrique du Sud dans le cadre d'un programme sandwich dans une faculté de médecine. Plus de 80% des diplômés travaillaient dans le secteur public (185/218, 82,6%), les chances diminuant de 27% pour chaque année après l'obtention du diplôme (odds ratio = 0,73; IC95%: 0,61 à 0,86, p <0,0001).

**Conclusions:**

Bien que la plupart des médecins restent au Malawi et dans le secteur public au cours de leur début de carrière, les risques de départ augmentent avec le temps. La majorité des diplômés à l'extérieur du Malawi poursuivent une formation en Afrique du Sud sous des restrictions de visa, reflétant ainsi l'impact positif de la formation postuniversitaire au Malawi. Les inquiétudes sur la rétention dans le secteur public sont réelles et nécessitent des explorations supplémentaires.

## Abstract (es)

**Objetivos:**

Existe preocupación sobre la migración de médicos de Malawi hacia países más desarrollados. El inicio de su carrera es particularmente vulnerable. Después de cambios políticos importantes, hemos examinado la retención dentro de Malawi y del sector público de médicos graduados recientemente.

**Métodos:**

Hemos obtenido, de la Facultad de Medicina de la Universidad de Malawi y del Ministerio de Salud de Malawi, datos sobre los médicos graduados entre el 2006 y 2012. Hemos utilizado la red de alumnos para triangular datos oficiales y hemos contactado directamente con aquellos graduados para los que no había datos o estos no eran precisos. Se utilizaron las pruebas de razón de momios (OR) y chi‐cuadrado para explorar posibles relaciones según año de graduación y género.

**Resultados:**

Seguimos a 256 graduados, con información completa para más del 90%. Casi un 80% de los doctores registrados estaban en Malawi (141/178, 79.2%), aunque la probabilidad de emigrar se doblaba con cada año después de la graduación (OR = 1.98, IC 95% = 1.54‐2.56, p <0.0001). De los 37 graduados que estaban fuera de Malawi (14.5%), 23 (62.2%) estaban formándose en Sudáfrica bajo un programa colaborativo de la Facultad de Medicina. Más del 80% de los graduados estaban trabajando en el sector público (185/218, 82.6%), con una probabilidad que disminuía un 27% por cada año transcurrido de la graduación (OR = 0.73, IC 95% = 0.61‐0.86, p < 0.0001).

**Conclusiones:**

Mientras que la mayoría de los doctores se quedan en Malawi y en el sector público al comienzo de su carrera profesional, la probabilidad de irse aumenta con el tiempo. La mayoría de los graduados que estaban fuera de Malawi estaban formándose en Sudáfrica con restricciones de visado, reflejando el impacto positivo de una formación de postgrado en Malawi. Las preocupaciones sobre el abandono del sector público son válidas y requieren de más trabajo exploratorio.

## Introduction

There have been longstanding concerns over the migration of Malawian medical doctors to high‐income countries (Muula & Broadhead 2001; Broadhead & Muula 2002; Joint Learning Initiative 2004; Record & Mohiddin 2006; Muula & Panulo 2007; Mills *et al*. 2011). In the past, medical students were sent abroad for training, most commonly to the United Kingdom, South Africa and Australia (Muula & Broadhead 2001; Broadhead & Muula 2002). The finding that a large majority were remaining outside Malawi after completion of training combined with continuing reliance on Western doctors to staff key clinical services led to the establishment of the first medical school in Malawi in 1991 (Muula & Broadhead 2001; Broadhead & Muula 2002; Zijlstra & Broadhead 2007). The curriculum at the University of Malawi College of Medicine (COM) had an emphasis on community health, aiming to sensitise students to conditions facing the majority of Malawi's population (Muula & Broadhead 2001; Broadhead & Muula 2002; Zijlstra & Broadhead 2007; Mullan *et al*. 2010). Since then, the number of graduates has increased gradually from 12 in 1992 to 47 in 2012 (Zijlstra & Broadhead 2007). However, the retention of Malawi‐trained graduates in Malawi has not been a resounding success. A tracing study in 2006 found that 40% of all graduates since 1991 were working or training abroad, with nearly half in the United Kingdom (Zijlstra & Broadhead 2007). This echoes findings from other sub‐Saharan countries (Hagopian *et al*. 2004; Mullan 2005; Clemens & Pettersson 2008), with a 2013 study finding emigration of doctors to the United States from a number of sub‐Saharan African countries had risen over the last decade (Tankwanchi *et al*. 2013).

In 2004, the assessment from Malawi's Ministry of Health (MoH) that the health worker situation was ‘critical [and] dangerously close to collapse’ led to a 6 year Emergency Human Resources Programme with major support from development partners (McCoy *et al*. 2008; Management Sciences for Health 2010). The ratios of health workers to population at that time were some of the worst in the world, with only 1.1 doctors per 100 000 people. Of the then 27 districts in Malawi (now 28), ten were without a MoH doctor and four without any doctor at all (Ministry of Health 2004). The goal of the programme was to increase levels of key health professionals to those of neighbouring Tanzania. Implemented measures included a 52% salary top‐up (nearer 30–35% after tax), other financial incentives such as continuation of free accommodation and transport to work (although a planned rural bonus was never implemented) and tripling the number of medical students at COM (Palmer 2006; Manafa *et al*. 2009; Management Sciences for Health 2010). These incentives were offered to all doctors employed in the public sector, and the entire programme costs approximately USD 30 million for all targeted health workers (Management Sciences for Health 2010). A final evaluation of the programme in 2010 determined that if all doctors trained had remained in the country, the doctor‐to‐population ratio should now be just below that of Tanzania at 2.03 per 100 000. No data were available at that time, however, to assess emigration of doctors (Management Sciences for Health 2010).

Another major policy change in 2004 was the introduction of postgraduate training at the now well‐established COM, again with support from development partners (Zijlstra & Broadhead 2007). This is a 4‐year Master of Medicine (MMed) degree that qualifies the candidate for registration as a specialist with the Medical Council of Malawi. In 2013, there were 27 specialist trainees enrolled across six specialties. For most specialties, this training is undertaken as a two‐part ‘sandwich’ programme: candidates initially work in Malawi for at least 2 years and then spend 18–24 months in South Africa to broaden trainees’ experience (Zijlstra & Broadhead 2007; Sawatsky *et al*. 2014). Moreover, visa restrictions negotiated with the South African authorities impede overstay of specialist trainees after their period of training, thus encouraging the return of trainees to Malawi (Zijlstra & Broadhead 2007). Other sub‐Saharan African countries such as Kenya and Tanzania also receive trainees, who complete all training in these countries without any bilateral agreements.

In the light of these significant changes in the educational and working environment of Malawian doctors, it is a pertinent time to update the 2006 tracing study by investigating the location of recent graduates (Zijlstra & Broadhead 2007) by investigating the location of recent graduates. Early career has been shown to be one of the most vulnerable times for migration of health professionals, with older graduates more effectively retained by ‘stick’ factors such as career establishment, assets acquirement and marriage that overcome strong push or pull factors for migration (Padarath *et al*. 2003; Mandeville *et al*. 2012; Tjadens *et al*. 2012). We examined the retention rate of Malawian doctors who graduated between 2006 and 2012.

### Overview of the health and medical education systems in Malawi

The provision of public health services is split two to one between the MoH and the Christian Health Association of Malawi (CHAM) a coalition of church organisations (Management Sciences for Health 2010). The Ministry of Health pays all staff salaries for Malawian nationals in CHAM facilities to facilitate the provision of health services in rural areas, where 84.0% of the population reside (2012 data, Global Health Observatory 2014). There are two major hospitals (Queen Elizabeth Central Hospital in Blantyre and Kamuzu Central Hospital in Lilongwe), two other tertiary hospitals (Mzuzu and Zomba Central Hospitals), 27 district hospitals and 23 CHAM hospitals.

After 5 years of medical education, graduate doctors must complete an 18‐month internship at either of the two major hospitals to be eligible for registration with the Malawi Medical Council. They are then allocated to a district as a district medical officer (DMO, supervising clinical care at district hospitals) or the more senior district health officer (DHO, responsible for district health programmes). Alternatively, they remain in tertiary hospitals as medical officers attached to a specialty department (Zijlstra & Broadhead 2007). After registration, doctors are able to work outside MoH facilities, for example for CHAM, non‐governmental organizations (NGO), private companies or research organizations.

## Methods

### Data collection

Data were collected as part of a research programme on the effectiveness of incentives to retain doctors in Malawi. For the purposes of this study, we sought information on four variables: (i) current job; (ii) location; (iii) currently in postgraduate training; and (iv) if so, funder of training. For a fifth variable, sector of work, we categorised participants based on their current job or postgraduate funder from the following: government, CHAM, research or teaching institutions, private organisations or NGO.

The COM Registry provided the names of those who graduated between academic years 2006 and 2012. As our primary interest was retention of Malawian graduates within Malawi, we excluded those graduates who were not Malawian citizens. We also excluded those who completed some or all of their undergraduate training outside Malawi as their career choices after graduation might differ systematically from those training in Malawi.

We reviewed three sources of secondary data to build up information on graduates: current location of all doctors in government jobs (provided by MoH), doctors in postgraduate training funded by government scholarships (MoH) and doctors registered for a COM postgraduate degree (COM).

As the first two data sets were found to be affected by time lags in updating official information, we triangulated these sources with information known to the research team through the alumni network. The alumni network for this cohort is very small, with an average of 37 graduates per year, and includes two members of the research team.

Finally, for those variables where there was a level of uncertainty or no information available, we contacted graduates directly through a combination of telephone, email and social networking sites using contact details obtained through the alumni network. For those graduates who did not reply, we collected data by proxy by questioning peers in the same year and triangulating amongst responses.

### Participants

**Figure 1:**
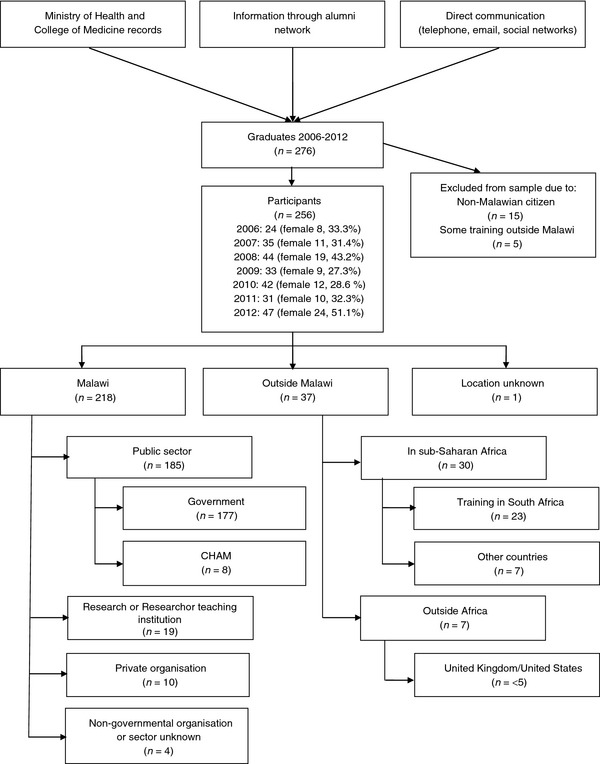
Flow of participants through study and overall distribution of graduates.

There were 276 Malawian graduates from COM between 2006 and 2012, with a mean of 36.6 per year. We excluded 15 graduates (5.4%) as non‐Malawian citizens and five (1.8%) who completed some undergraduate training outside Malawi (Figure 1). Of the 256 remaining graduates, of whom 36.3% were female (93/256), we directly contacted 54 (21.1%) to verify uncertain variables or obtain missing data. Complete information (data on all five variables) was obtained for more 91.0% (233/256) and at least partial (four or fewer variables) for all graduates. Missing data largely pertained to funder of postgraduate training and associated sector of work (Table 1). Data on graduates were obtained directly for 93% (237/256) and by proxy for 7.1% (18/256). There were no deaths reported in this cohort.

**Table 1:** Distribution of graduates by year

Year	Graduates (*n*)	In Malawi		Outside Malawi (%)	In postgraduate training (%)	Training in South Africa (%)
		Public sector (%)	Rural areas (%)			
2006	24	13 (54.2)	5 (20.8)	9 (37.5)	14 (58.3)	7 (29.2)
2007	35	15 (42.9)	<5 (<14.3)	12 (34.3)	22 (62.9)	9 (25.7)
2008	44	28 (63.6)	12 (27.3)	8 (18.2)	27 (61.4)	6 (13.6)
2009	33	24 (72.7)	11 (33.3)	6 (18.2)	15 (45.5)	1 (3.0)
2010	42	30 (71.4)	17 (40.5)	<5 (<11.9)	5 (11.9)	0
2011	31	28 (90.3)	10 (32.3)	0	0	0
2012	47	47 (100)	0	0	0	0
Total	256	185 (84.9)	<60 (<23.4)	<40 (<15.6)	83 (32.4)	23 (9.0)
Missing data*		5 (2.0)		1 (0.4)	1 (0.4)	

Categories are not mutually exclusive. Row percentages refer to number of graduates for that year, except for missing data which is out of total number of graduates. *Other missing data: Current job = 4 (1.6%); Funder of postgraduate training = 18 (7.0%).

### Data analysis

Data were entered and cleaned in Microsoft Excel 2010. Due to the small numbers involved, some results have been aggregated into larger categories or not specified if less than five to preserve participant anonymity. For the purposes of this analysis, we aggregated government and CHAM into ‘public sector’. Pre‐specified statistical tests were carried out in Stata version 12.0, including logistic regressions to analyse trends by year of graduation and chi‐squared tests to examine outcomes by gender.

### Ethics approval

Ethics approval was obtained from the COM Research and Ethics Committee of the University of Malawi and the London School of Hygiene and Tropical Medicine.

## Results

Eight‐five percentage of recent graduates (218/256, 85.2%) were still in Malawi (Figure 1). If we exclude interns due to the mandatory period within Malawi to register with the Malawi Medical Council, then nearly 80% (141/178, 79.2%) of registered medical practitioners are still in Malawi. Only seven graduates (7/256, 2.7%) were outside Africa, with fewer than five in the UK/USA. Overall, fewer than five graduates were working outside of Malawi and not in postgraduate training (<5/256, <2.0%).

Of the 30 graduates in other sub‐Saharan African countries (30/256, 11.7%), three quarters (23/30, 76.7%) were undertaking the South African component of their MMed postgraduate training. Fewer than five graduates (<5/30, <16.7%) were training in sub‐Saharan African countries without visa restrictions. The odds of a doctor being outside Malawi doubled for each year after graduation (odds ratio (OR) = 1.98, 95% confidence intervals (CI) = 1.54–2.56, *P* < 0.0001, Table 1); however, these odds fell if those training in South Africa are excluded (OR = 1.45, 95% CI = 1.06–2.00, *P* = 0.021).

Nearly 85% of recent graduates were working or training in the public sector in Malawi (185/218, 84.9%). The odds of a doctor working in the public sector declined by 27% for each year after graduation (OR = 0.73, 95% CI = 0.61–0.86, *P* < 0.0001, Table 1). The largest attrition from the public sector (MoH or CHAM) within Malawi was to research organisations, with one in 12 graduates working as research officers (18/218, 8.3%). Ten graduates (4.6%) were working for private facilities or commercial organizations and fewer than five (<5/218, 2.3%) for non‐governmental organisations (Figure 1). Men were no more likely to be working outside the public sector than women (16.6% *vs*. 12.3%, OR 1.41, 95% CI 0.67–3.02, *P* = 0.374).

**Figure 2:**
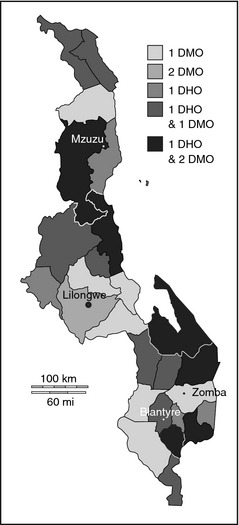
Distribution of graduates working at district level. Map denotes districts of Malawi. DMO, District Medical Officer; DHO, District Health Officer.

Figure 2 maps the distribution of 2006–2012 graduates working at district level. While pre‐2006 graduates would also be working at district level, 21 doctors from this cohort were working as DHOs and 30 as DMOs, with seven district hospitals benefitting from two graduates as DMOs. If we define an urban location as any of the four major cities in Malawi (Lilongwe, Blantyre, Mzuzu or Zomba) and a rural location as elsewhere, as well as exclude interns due to their mandatory training at the two urban teaching hospitals, then nearly a third of recent graduates (27.5%, 57/207) in Malawi are based in rural areas (Table 1). Men were significantly more likely to be based in rural areas compared to women (34.3% *vs*. 12.5%, OR= 3.67, 95% CI 1.73–7.74, *P* = 0.0002).

Overall, one third of graduates were in postgraduate training (83/256, 32.4%), with doctors twice as likely to be in training for every year after graduation (OR = 2.18, 95% CI = 1.78–2.68, *P* < 0.000, Table 1). Over half of graduates in training (55.4%, 46/83) were on government scholarships, either funded directly by the Malawian government or by development partners. One in 10 trainees (9/83, 10.8%) were self‐funding or had procured private scholarships, including all graduates training outside Africa. Eight trainees (9.6%, 8/83) were being sponsored by CHAM or research organisations, with no trainees funded by private organisations. The odds of being in training were not significantly different between graduates in the public or other sectors (30.6% *vs*. 29.6%, OR = 1.05, 95% CI 0.49–2.26, *P* = 0.904). Those on government scholarships were less likely to be from.

## Discussion

We traced 256 doctors from a seven‐year cohort of graduates at Malawi's only medical school, finding that nearly 80% of registered doctors were located in Malawi. The odds of emigrating, however, doubled for each year after graduation. Of the graduates outside Malawi, over four fifths were in postgraduate training with over 60% in South Africa. Over 10% of postgraduate trainees were self‐ or privately funded, including all those training outside Africa. Within Malawi, 85% of graduates were working in the public sector. The odds of leaving the public sector increased with time, with the largest attrition to research organisations. Graduates were well distributed at district level, with all districts being served by at least one recent graduate.

There is a high retention of medical graduates in Malawi during the early part of their career. This study refutes findings from a survey of medical students at COM in 2008 that found nearly 40% of medical students intended to work or train outside Malawi soon after graduation (Mandeville *et al*. 2012) and corroborates a general perception of fewer doctors emigrating identified through qualitative work (Bailey *et al*. 2012). It also contrasts with findings from other sub‐Saharan countries, where graduates between 2005 and 2008 stayed on average just 1.3 years in their country of training before emigrating to the USA (Tankwanchi *et al*. 2013).

While retention soon after graduation was high, the odds of emigration increased with time. This may reflect growing disillusionment as work experience increases or the time required to identify opportunities outside of Malawi. There is reason to believe, however, that the current trend is less pessimistic than first appears. The largest group of graduates outside Malawi were those training in South Africa as part of the COM sandwich programme, with bilateral agreements in place to encourage return to Malawi, and fewer than five graduates were in the United Kingdom or United States. This is a more positive situation than previously and provides support for the positive impact of the COM postgraduate training programme. In comparison, the study by Zijlstra and Broadhead (Zijlstra & Broadhead 2007) traced all graduates from 1991 to 2006. While retention rates by year of graduation were not available, they found 59.7% of registered doctors (123/206) in Malawi, with a third of emigrants unlikely to return (28.9%, 24/83) and nearly half in the United Kingdom (40/83, 48.2%). Further follow‐up of the cohort studied here is required to ascertain whether emigration continues to rise with age and whether training in Malawi or sub‐Saharan Africa decreases the likelihood of emigration after completion of specialist training.

The importance of postgraduate training is underlined by one third of early graduates being in training, with doctors twice as likely to be in training for every year after graduation. Specialisation is seen as integral to career progression by medical students and early graduates both in Malawi (Bailey *et al*. 2012; Mandeville *et al*. 2012) and other sub‐Saharan African countries (Burch *et al*. 2011), with the quality and availability of postgraduate training a primary concern (Burch *et al*. 2011; Bailey *et al*. 2012; Sawatsky *et al*. 2014). There has been some increase in the number of government scholarships: one pooled funding scheme (the National AIDS Commission) provided 5 years of continuous scholarships, and new funding has been obtained for obstetrics and gynaecology and surgery training. Yet postgraduate training has not kept pace with the expansion in medical student numbers under the Emergency Human Resource Programme (Management Sciences for Health 2010). In a vacuum of training opportunities in the public sector, graduates are likely to look elsewhere for funding (Bailey *et al*. 2012). One in 10 postgraduate trainees was self‐funding or on private scholarships, including all those who were training outside Africa. Some may have deliberately sought to train outside Africa, with ambiguous feelings held towards the COM postgraduate programme by medical students (Sawatsky *et al*. 2014) and nearly 90% intending to specialise abroad (Yeganeh‐Arani *et al*. 2012). Yet it is likely that some graduates, who would have preferred to have trained within Malawi, were unable to do so due to difficulty in obtaining government scholarships. A whole‐career approach should be taken to medical training with postgraduate training increased proportionally to pre‐service training (Mullan *et al*. 2010; Greysen *et al*. 2011), in order to maximise the return on government investment into medical education (previously estimated at USD57 000 per doctor) (Muula & Panulo 2007).

Concerns over graduates leaving the public sector have some basis, however, with the odds of attrition increasing with time after graduation. Possible reasons include differences in remuneration, prospects of postgraduate training scholarships, working conditions or career progression. Salaries are certainly higher in other sectors (Muula & Maseko 2005; McCoy *et al*. 2008), and those working for research or private organisations in this cohort earned three times the salary of graduates in the public sector with a mean of MK344 000 per month [USD869] *vs*. MK114 000 [USD288] (unpublished data Mandeville, KL). This takes into account the salary top‐up under the Emergency Human Resources Programme, but not free housing/transport or supplements such as *per diems* for meeting attendance. Yet previous qualitative work found little interest in working for non‐governmental or private sector organisations amongst Malawian medical students and interns, with a perception of ‘being better looked after’ in the public sector (Bailey *et al*. 2012). For example, private sector employees are not eligible for government training scholarships: making the public sector attractive as a route towards professional development (Muula & Maseko 2005; Sawatsky *et al*. 2014). Although no private organisations were funding trainees in this cohort, we actually found that graduates in other sectors were no less likely to be in training than those in the public sector. Further qualitative work is required to delineate the reasons behind this movement out of the public sector.

Research organisations were the largest absorber of graduates leaving the public sector, compared to private or non‐governmental organisations. While such jobs may be time limited and confer important additional skills, graduates may be ‘poached’ during a period where the government is still recouping their investment into training through public sector service. There may, of course, be later re‐entry into the public sector, although it has been reported that there are barriers to such re‐employment in Malawi (Muula & Maseko 2005). Furthermore, while more graduates leave the public sector with time, attrition may tend towards a steady state: Zijstra and Broadhead also found 79.6% (98/123) of graduates working for the public sector over a 15‐year period (Zijlstra & Broadhead 2007).

Nearly a third of recent graduates are based in rural areas, compared to 18.3% of all registered doctors in Malawi in 2009 (Africa Health Workforce Observatory 2009). While previously doctors at district level were few and usually working in isolation (Muula & Maseko 2005), now six districts have a team of one DHO and two DMOs from recent graduates alone. It appears that the policy of posting newly registered doctors to districts has achieved good coverage of rural areas by recent graduates, despite the lack of rural allowances (Management Sciences for Health 2010). Yet the majority of recent graduates are still concentrated in the two major hospitals, where most specialists and training capacity reside. As the number of specialists increases, an active policy of building up the training capacity of the smaller central hospitals to continue redistribution of graduates outside the two major cities will be important. Women were significantly less likely to be working in rural areas than men, which is corroborated by findings from rural practice in other countries (Laven & Wilkinson 2003; Matsumoto *et al*. 2008). This may reflect demand or supply side gender bias, with women less likely to be allocated to rural areas, more likely to petition to remain in urban areas or more likely to leave rural jobs than men.

These results strengthen a field characterised by a paucity of research, with 47 of 58 sub‐Saharan African medical schools reporting no formal tracking of graduates (Mullan *et al*. 2010). While some sub‐Saharan countries utilise medical registration and licensing requirements as a tracking mechanism, these systems suffer from a lack of enforcement, impeding accurate follow‐up (Chen *et al*. 2014). To overcome the fragmented health workforce records found in many low‐resource settings (Riley *et al*. 2012; Chen *et al*. 2014), we cross‐validated official records with more current information obtained through the alumni network. This enabled us to build up a comprehensive database on recent graduates, with complete information for over nine in 10 graduates and at least partial information on all graduates. There is a risk that the alumni data could be less accurate than official records; however, these were triangulated between two researchers who interact with the alumni community daily and graduates were contacted directly if there was any uncertainty. Moreover, it would be short‐sighted to favour official records in a weak institutional environment over the real‐time information based on natural social groupings provided by networking sites such as Facebook. Indeed, the power of social media is being harnessed for information gathering and dissemination in many areas of health policy (Merchant *et al*. 2011; Thackeray *et al*. 2012; Mandeville *et al*. 2014), and five sub‐Saharan African medical schools are now using social media to maintain contact with graduates (Chen *et al*. 2014). We report here a low‐cost yet effective method for tracking graduates that leverages the strengths of a small alumni community, which could be considered in similar settings in order to obtain timely data to inform health workforce policy.

Limitations of our design include the lack of data collected on sociodemographic variables, which may influence later career choices. For example, a study following up the first 22 years of graduates over 22 years from Nepal's public medical school found that a rural birthplace was associated with work in rural areas and paramedical training before medical school was significantly associated with lower migration outside Nepal (Zimmerman *et al*. 2012). We also could have followed up a larger cohort of graduates in order to capture more graduates undertaking and completing specialist training. However, this would have lost the homogeneity of experience in terms of training and working environment that allows us to draw tentative conclusions on migration decisions in the absence of period effects. While obtaining data through proxies may be criticised for possible inaccuracy, this was required for less than one in 10 graduates and is a pragmatic approach employed by other studies in low‐resource settings (Zimmerman *et al*. 2012). Finally, this study reflects just one point in time, whereas the job choices of graduates are likely to be dynamic, particularly during early career. Further follow‐up of this cohort would enable investigation of movement between sectors, including the rate of return after completion of training.

## Conclusions

While most doctors remain in Malawi and the public sector during their early career, the odds of leaving both increase over time. The majority of those outside Malawi are training as part of a sandwich programme, reflecting the positive impact of postgraduate training in Malawi. Postgraduate training capacity should be increased proportionally to undergraduate training. Concerns over attrition from the public sector are valid, requiring further exploratory work.
